# Sequence Specific Motor Performance Gains after Memory Consolidation in Children and Adolescents

**DOI:** 10.1371/journal.pone.0028673

**Published:** 2012-01-19

**Authors:** Shoshi Dorfberger, Esther Adi-Japha, Avi Karni

**Affiliations:** 1 The Edmond J. Safra Brain Research Center for the Study of Learning Disabilities and Learning Disabilities Department, University of Haifa, Haifa, Israel; 2 The School of Education, Bar Ilan University, Ramat-Gan, Israel; The University of Western Ontario, Canada

## Abstract

Memory consolidation for a trained sequence of finger opposition movements, in 9- and 12-year-old children, was recently found to be significantly less susceptible to interference by a subsequent training experience, compared to that of 17-year-olds. It was suggested that, in children, the experience of training on any sequence of finger movements may affect the performance of the sequence elements, component movements, rather than the sequence as a unit; the latter has been implicated in the learning of the task by adults. This hypothesis implied a possible childhood advantage in the ability to transfer the gains from a trained to the reversed, untrained, sequence of movements. Here we report the results of transfer tests undertaken to test this proposal in 9-, 12-, and 17-year-olds after training in the finger-to-thumb opposition sequence (FOS) learning task. Our results show that the performance gains in the trained sequence partially transferred from the left, trained hand, to the untrained hand at 48-hours after a single training session in the three age-groups tested. However, there was very little transfer of the gains from the trained to the untrained, reversed, sequence performed by either hand. The results indicate sequence specific post-training gains in FOS performance, as opposed to a general improvement in performance of the individual, component, movements that comprised both the trained and untrained sequences. These results do not support the proposal that the reduced susceptibility to interference, in children before adolescence, reflects a difference in movement syntax representation after training.

## Introduction

In a recent study we found that motor memory consolidation, in 9 and 12-year-old children, was significantly less susceptible to interference by a subsequent training experience compared to that of 17-year olds [1]. One proposal to explain these results was that, in children, the experience of training on a given sequence of finger movements may affect the performance speed of each movement element rather than the syntactic rule which has been implicated in the learning of the task in adults [2]–[6]. Thus, the initially trained movement sequence and the subsequently trained (‘interference’) sequence would constitute, in children, two instances of training on a similar set of movements rather than two different sequences. This would result in less competition between the two sequences (i.e., representational overlap) and less interference [7]–[9]. A testable corollary of this proposal is the hypothesis that, in children, training on one sequence would result in enhancement of the training experience on a subsequent movement sequence if both sequences are composed of the same component movements. In young adults, however, it was previously shown that the gains in performance retained after a training experience are sequence specific, i.e., cannot be expressed in the performance of a different sequence; even a new sequence composed of the same component movements of the trained sequence (e.g. [4]–[5]).

The ability to transfer performance gains is a measure of the possible advantage incurred by past experience (i.e., training or practicing) as reflected in the performance of a familiar task under new circumstances, or in performing a related but novel task. The importance of tests to assess transfer relates to the possibility that the analysis can provide important constraints for localizing the level of representation of the trained task in the brain, i.e., in probing where, in terms of brain representations, training dependent changes took place (e.g. [10]–[12]). The logic behind this approach is that partial or lack of transfer of the learned knowledge to novel conditions is an indicator that learning has occurred at a neural level wherein critical aspects of the novel conditions are represented separately from the parameters of the original training conditions [13]–[16]. For example, gains in motor performance that do not transfer from a trained to an untrained hand can be taken as indicating a learning-dependent change within a motor representation in which the neuronal population represents movement of one effector but not of its opposite (a lateralized representation) [3]–[4].

Previous studies on transfer effects in the FOS learning task, in adults, indicated that, in adults, there was transfer from the trained hand to the untrained hand at the completion of one training session [4]–[5], [17] and after a 48 hours consolidation phase [3]–[4], [17] but not after multi-session training [2]–[5], [16], [18]. Inter-manual transfer (effector invariant learning) was found, in early stages of practice, in other tasks as well, in both adult humans and monkeys [16], [18]–[20]. However, the practice related gains, in adults, were found to be sequence-of-movement specific; significant differences were found between the trained and reversed sequence in both speed and accuracy [2], [4]–[5], [17].

There are no published data on hand or sequence specificity of the practice dependent gains in the FOS task, in children. The aim of the current study was to test whether the gains attained in the performance of the FOS task after a single training session, and a 48 hours memory consolidation interval, can be transferred to a different arrangement of the trained movement components in preadolescents as compared to 17-year-olds; the latter would presumably show the adult pattern of sequence specific gains in performance [1].

## Methods

The performance of a trained movement sequence executed by the trained (left, non-dominant) hand of participants, from three age-groups, was compared to the performance in three untrained conditions (transfer conditions) at 48 hours after a single training session. Three transfer conditions were tested: a) the reversed sequence (identical component movements arranged in the reversed order) performed by the trained, left, hand (LR); b) the trained sequence performed by the untrained, right, hand (RT) condition, and c) the reversed sequence performed by the right hand (RR). The data were obtained from participants who took part in a motor memory consolidation study [1].

### Participants

Sixty-two participants, from three age-groups (9, 12, and 17-year-olds), took part in the experiment. Group 1 (age 9) was comprised of 11 girls and 10 boys, Group 2 (age 12) of 10 girls and 11 boys, and Group 3 (age 17) of 10 girls and 10 boys. Participants were recruited from schools in a suburban neighbourhood of middle to high socio-economic level. Participants were right-handed, had no outstanding medical conditions that could impair fine motor performance, reported at least 6 hours of sleep per night, and had no sleep–wake-cycle disruptions. All participants attended elementary, middle and high school in accordance with their age. Inclusion criteria included remembering 5/5 digits in a forward digit span test in order to ensure the explicit, short-term memory retention of a 5-element sequence. The experiment was approved by the University of Haifa human experimentation ethics committee and the Israeli Ministry of Education; informed parental consent was obtained.

### The task

**Figure 1:**
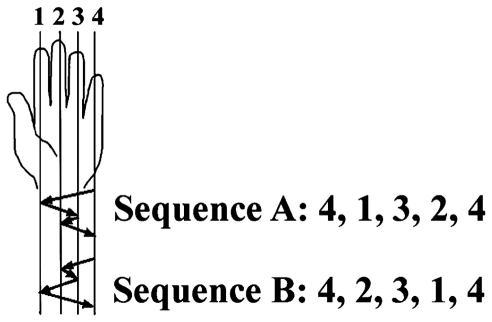
The finger-to-thumb opposition task. The two sequences were matched for number of movements per digit and mirror-reversed in relation to each other (in terms of order).

The motor task was the finger-to-thumb opposition sequence (FOS) learning task as previously described [1] (Figure 1). Two sequences of equal length and complexity were used, each being the reverse of the other. These were (numbering the fingers 1-4, with 1 designating the index finger and 4 the little finger): 4-1-3-2-4, or 4-2-3-1-4. Each participant was randomly assigned one of the sequences for training. Participants were instructed to oppose the fingers of the left (non-dominant) hand to the thumb in the given 5 movement sequence “as quickly and accurately as possible” (Figure 1). The participants performed the instructed movements while lying supine with the hand positioned on their chest, with the elbow flexed, in direct view (palm-facing) of a video camera, to allow recording of all finger movements. Participants were instructed to look up so that visual feedback was not afforded.

### Procedure

The experiment included four videotape-recorded sessions. The three sessions occurred in three successive days. In the first session (day 1) each participant underwent training that consisted of 20 consecutive blocks, each block constituting a 30 sec interval, wherein the finger opposition movement sequence was repeatedly performed. The initiation of each block and its termination were cued by an auditory signal. Participants were instructed to tap the movement sequence continuously until given the stop signal, and if any error occurred, to continue with the task without pause, as smoothly as possible. The breaks between blocks were no longer than 20 seconds. Before each block the participants repeated the assigned sequence three times freely, as a means for maintaining their attention on the task, and as a practice run. No feedback on any performance measure was provided, besides general encouragement. In the second session (day 2) i.e., 24 hours after the first session, participants were tested in 4 successive blocks identical in content and procedure to the blocks performed in the first session. In the third session (day 3, 48 hours post-training) participants were tested in 4 successive blocks identical to the blocks used in the first session performed separately in each hand and 4 consecutive blocks of its reversed order (a sequence containing the same five movements in the opposite order) performed separately in each hand.

The trained condition (T) was always performed first. Next, the performance of the trained sequence by the untrained hand (RT condition) was tested. This was followed by tests of the reversed sequence performed by either the right hand (RR) or the left hand (LR). The testing of the reversed, untrained, sequence was counter balanced across participants in a pseudorandom manner, with half the participants first tested with the untrained hand, while the others were first tested with the trained hand.

Two dependent variables were measured, separately, for each test block (30 sec interval): a) performance speed – the mean number of correct sequences tapped; b) accuracy – the mean number of sequencing errors (wrong finger opposition order). In the statistical analysis the average for each set of 4 test blocks, at each time-point, was used. The age-group constituted a between-subject factor, while time-points (*end, 48h-post*) and test conditions (T, RT, RR, LR) were considered as within-subject factors in the analyses of variance. Therefore, a mixed model ANOVA was used (rm-ANOVA). Scheffe's method was used to account for multiple comparisons.

## Results

**Figure 2:**
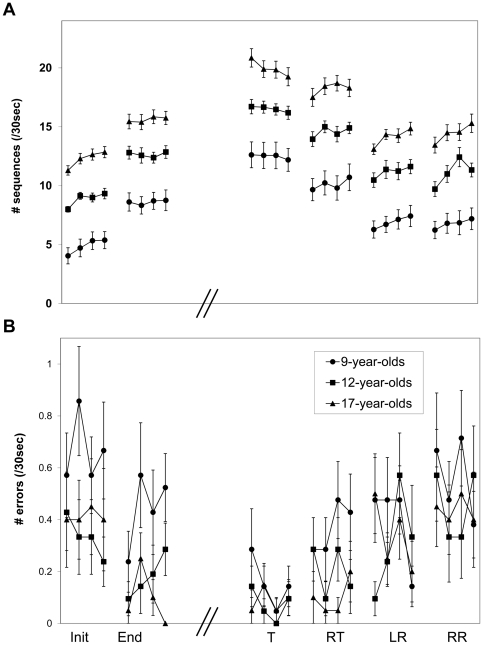
Retention and transfer after a single training session. Shown is performance on the trained and transfer conditions at 48 hours post-training (T, trained; LR, left reversed; RR, right reversed; RT, right trained). Bars –Standard Error.

The training experience was highly effective in all three age groups. As previously reported [1] there were robust gains in performance speed with no reduction in accuracy across the training session, in all three age groups. There were also additional, highly significant, gains in both speed and accuracy across the post-training interval (delayed consolidation phase gains). A comparison of performance by the end of the training session (*end*) to the performance attained in the 48 hours post-training test showed robust gains in speed (*F*(1,59) = 179.29, P<.001) as well as a significant increase in accuracy (*F*(1,59) = 10.01, P<.001) in all three age groups. There was no interaction for *time-points* and *age-groups* for speed (*F*(2,59) = 0.28, P = .76), but there was a significant interaction for *time-points* and *age-groups* for accuracy (*F*(2,59) = 3.51, P<.05). In order to explain the latter interaction, paired t-tests were used to compare the number of errors at the end of the training session to the number of errors committed at 48 hours post-training for each group separately. On average, there were small improvements in all three groups but this change was statistically significant only in the youngest age-group (t(20) = 2.96, p<0.05; t(20) = 1.48, t(20) = 1.48, P = .15; t(19) = 0.33, P = .75 in the 9, 12 and 17-year-olds, respectively) (Figure 2).

In terms of speed, there was a significant *age-group* effect (*F*(2,59) = 30.65, P<.001) with better performance in the 17-year-olds compared to the 12-year-olds, and with better performance in the 12-year-olds compared to the 9-year-olds (Scheffe<.001, Figure 2). In terms of accuracy, there was a significant age-group effect (*F*(2,59) = 4.09, P<.05) with better performance in the 17-year-olds compared to the performance of the 9-year-olds (Scheffe<.05).

To test the ability to transfer the training related performance gains, the performance (speed and accuracy) in the 3 transfer conditions was compared to the performance of the trained movement sequence when performed with the trained hand. A rm-ANOVA was run for speed and again for accuracy with the three *age-groups* (9, 12, 17-year-olds; as a between-subject factor)×four *conditions* (the trained sequence by the trained hand (T), the trained sequence in the right hand (RT), the reversed, untrained, sequence performed by the left hand (LR); the reversed sequence performed by the right hand (RR); as a within-subject factor). There was a main effect of *condition*, for both the number of sequences (*F*(3,177) = 167.66, *P*<.001) and the number of errors (*F*(3,177) = 18.18, *P*<.001) but no significant interaction for *condition* and *age-group* (*F*(3,177) = 0.32, *P* = .93; *F*(3,177) = 0.55, *P* = .77; speed and accuracy respectively) indicating a similar limitation in the ability to transfer the gains to novel task conditions in all age-groups. Also, there was a significant age effect for speed (*F*(2,59) = 35.96, *P*<.001) with better performance in the 17-year-olds compared to the 12-year-olds and with better performance in the 12-year-olds compared to the 9-year-olds (Scheffe<.001, Figure 2). There was no significant age effect for accuracy (*F*(2,59) = 1.41, *P* = .25).

To further assess the ability to transfer the gains from the trained movement sequence to the performance in each of the three transfer conditions additional rm-ANOVAs were run.

### Trained sequence specificity in the trained hand

Significant sequence specificity was found when the trained and the reversed sequence were tested in the left, trained, hand in all three age groups (2 *conditions* (T, LR)×3 *age-groups* (9, 12, 17-year-olds)). Performance speed in *T* was significantly better than in *LR* (*F*(1,59) = 230.53, P<.001) with no interactions for *condition* and *age-groups* (*F*(2,59) = 0.15, P = .86). In addition, there were significant age-group effects (*F*(2,59) = 34.83, P<.001) with better performance in the older age-groups. In terms of accuracy, there was again a significant main effect of *condition* (*F*(1,59) = 31.19, P<.001) and no interactions for *condition* and *age-groups* (*F*(2,59) = 0.01, P = .99) as well as no significant differences between the three age-groups (*F*(2,59) = 0.78, P = .46).

### Trained sequence specificity in the untrained hand

Transfer from the trained to the untrained sequence was very limited also in the untrained hand. The performance of RT was significantly better than that of RR. Moreover, sequence specificity was of a similar magnitude in the three age-groups. For performance speed, there was a significant main effect of *condition* (*F*(1,59) = 157.98, P<.001) with no significant interaction for *condition* and *age-groups F*(2,59) = 0.24, P = .79). There was a significant age-group effect (*F*(2,59) = 33.62, P<.001) with better performance in the older age-groups. For accuracy, there was a significant main effect of *condition* (*F*(1,59) = 19.83, P<.001) with no interaction for *condition* and *age-groups F*(2,59) = 0.53, P = .59) and no significant differences between the three age-groups (*F*(2,59) = 1.43, P = .25).

### Transfer of gains for trained sequence between the hands

When the performance of the trained sequence by the two hands was compared (T, RT) there was a significant main effect of *condition F*(1,59) = 68.12, P<.001). As can be seen in Figure 2a, the performance of the trained hand was better than the performance of the right, untrained hand. There was no significant interaction for *condition* and *age-group* (2,59) = 0.62, P = .54) indicating that the limit on transfer between the two hands was of a similar magnitude in the three age-groups. Again, there was a significant *age-group* effect (*F*(2,59) = 27.33, P<.001) with better performance in the older age-groups. Similarly, for accuracy, comparing *T* to *RT* showed a significant main effect of *condition F*(1,59) = 5.75, P<.001) with the performance in the trained hand more accurate than the performance of the right, untrained hand (Figure 2b). There was no significant interaction for *condition* and *age-group F*(2,59) = 1.37, P = .26) and no significant difference between the three age-groups (*F*(2,59) = 2.69, P = .08).

### Novel sequence performance by the left vs the right hand

To indirectly test whether there was a difference between the two hands to begin with (i.e., before training), the performance of the untrained sequence by the dominant hand, at 48 hours post-training, and the initial performance before training, for the to-be-trained sequence by the non-dominant hand (*init*, Figure 2) were compared (RR compared to initial performance of T). This analysis, therefore, compared the initial performance of an untrained sequence by the two hands. Initial performance in the left hand for T was slower than that of the right hand for RR (a significant main effect of *condition* (*F*(1,59) = 51.91, P<.00)). There was no interaction of *condition* and *age-groups* (*F*(2,59) = 0.13, P = .88) but overall the performance of the older age groups was superior in both hands (a significant *age-group* effect (*F*(2,59) = 49.37, P<.001)). There were no significant differences in the number of errors committed in the initial performance of the novel movement sequences when the two hands were compared (*F*(1,59) = 0.02, P = .09). Also, the interaction of *condition* and *age-groups* was not significant (*F*(2,59) = 0.67, P = .52) and there were no significant group differences in accuracy (*F*(2,59) = 2.17, P = .12).

The apparent advantage of the right hand may have reflected hand dominance, i.e., the fact that all participants were right handed. Alternatively, the apparent right hand advantage may have resulted from a small but non-specific transfer effect with the performance in the RR condition reflecting and building on the prior experience of the left hand. To test this possibility, the performance of the reversed, untrained, sequence in the two hands was compared (LR compared to RR). Note that in this analysis the performance of both hands can build on the prior training experience afforded in the training session, two days previously, but because the order of testing the two hands was randomized across participants, in each age group, half the participants experienced the untrained, reversed, sequence first with the dominant hand and the other half with the non-dominant hand. As can be seen in Figure 2, there was no significant advantage of one hand over the other in speed (*F*(1,59) = 0.05, P = .83) but for accuracy, there was better performance of the left, trained, hand compared to the right hand (*F*(2,59) = 5.66, P<.05). There were no significant interactions of *condition* and *age-group* (*F*(2,59) = 0.41, P = .67; *F*(2,59) = 0.11, P = .89; speed and accuracy, respectively). In both hands, the older participants outperformed the younger ones in terms of speed (*F*(1,59) = 35.62, P<.00; Scheffe<.001) but not in terms of accuracy (*F*(1,59) = 0.46, P = .64).

## Discussion

The prediction that the ability to transfer the training-related gains in motor sequence performance would be different in children (before adolescence) and 17-year olds was not supported by our data. The performance of participants from the three age-groups, at 48 hours post-training, was significantly better for the trained sequence performed by the left (trained) hand compared to the reversed sequence performed by the left hand, in terms of both speed and accuracy. Moreover, while all three age-groups showed significant transfer of the training-related gains from the trained (left) to the right (untrained, dominant) hand, there was a clear advantage of performance for the trained movement sequence over the untrained sequence even when performed with the untrained hand. Thus, there was only minimal transfer of the practice related gains between the trained movement sequence and a motor sequence composed by the same opposition movements but arranged in a reversed order. The differences in the performance of the two sequences, in terms of speed and accuracy, clearly indicate sequence specific learning of the FOS task in children as well as in the 17-year-olds.

The overall pattern of limits on transfer is in line with the sequence specificity and effector independence of the gains reported for young adults by 24 to 48 hours after a single training session [3]–[5], [17]. Sequence specific learning has been consistently found even in 6 year olds [21]–[22]. Thus, the current results indicate that, as in adults, the gains attained after a single session of training and an effective consolidation interval, are specific for the trained order of movements (the trained sequence) and not for the rate at which the five sequence elements are executed *per-se* in pre-adolescents.

Although the gains attained in the left hand, for the trained movement sequence, showed significant transfer to the untrained hand, there was a clear limit also on the inter-manual transfer in all three age groups. The performance of the trained sequence in the trained hand was significantly better compared to the performance of the trained sequence in the untrained hand, indicating that the transfer of the gains to the untrained hand was incomplete. This incomplete transfer, however, was on a level very similar to that previously reported for young adults training in the FOS task [4].

The current results show that the small advantage of the second (right) hand in the performance of an untrained sequence, given the identical constraints on the order of task conditions, was of a similar magnitude in the three age-groups. There were no significant interactions of the test conditions and age-groups, indicating that in the three age-groups tested, the ability to transfer the sequence specific and possibly the non-specific gains between the left trained hand and the right untrained one did not significantly differ in the 9, 12 and 17 years old.

One cannot rule out the possibility that there was also some, minimal but significant, non-specific transfer of gains from the trained to the untrained hand. When comparing the two hands in the performance of a newly introduced sequence of opposition movements, the initial performance speed for the to-be-trained sequence in the left hand and the initial performance of the reversed (untrained) sequence in the right hand, there was a small but significant advantage for the latter hand, in all three age groups. The second (right) hand advantage was reflected only in the speed of performance but not in accuracy. The small advantage in speed may reflect an order-of-training effect, with the initial training experience of one hand conferring some advantage to the other hand even in the performance of a novel movement sequence (both sequences composed of the same component movements). This latter possibility was tested by comparing the two hands 48 hours after the initial training experience with the complementary movement sequence. There was no significant difference in the speed of performance of the reversed (novel) sequence by the two hands, but there was a small but significant advantage for the left hand, which does not support the notion of non-specific inter-manual transfer.

The latter results also do not support the notion that the second hand advantage was due, at least in part, to a hand dominance effect. Previous studies on right hand dominant young adults failed to show significant differences in the initial performance of the hands in the FOS task (e.g. [4]) or pegboard task (e.g. [19]). An asymmetry in sequence representation was suggested for learning with either the left or the right hand in the SRT task (e.g. [23]) although the two hands' initial performance was quite similar.

Limited transfer of training-dependent performance gains across motor effectors can be viewed in analogy to the well described phenomenon of visual field specific perceptual learning [12], [14]–[15], [24]. Note that there is good evidence supporting the notion that the structure of the training experience may constitute an important factor in determining the locus of the practice related changes subserving the acquired skill and therefore the profile of transfer ability may change under different practice schedules [4], [25]. In some perceptual discrimination learning protocols, specifically those involving training on more than one task condition within a given session [26] but also after a single short training experience [11] non-specific transfer of gains across visual field locations have been described. Further practice however, may diminish this initial non-specific aspect of a given skill [4], [11], [13], [18]. Although the transfer profile of the practice related FOS performance gains after multi-session training remains to be determined, the current results nevertheless show that at least after a single training session, and a memory consolidation interval [1], the ability of children to generalize the gains is as limited as that of adults. On the other hand, the tests of the three transfer conditions analysed in the current study clearly show that maturational factors may be at work, with superior performance in the transfer conditions as well as in the trained task, in the older age groups. The significant age-group effects for speed of performance indicated that performance was generally faster in the older age-groups compared to the younger participants. In terms of accuracy, the oldest age-group was more accurate than the youngest group by 48-hours post training in either the trained condition or the three transfer conditions.

The current analysis was undertaken to test the possibility that a difference in movement representation, before and after adolescence, may explain the finding [1] that motor memory consolidation for a trained sequence of finger opposition movements, in 9 and 12-year-old children, was significantly less susceptible to interference by a subsequent training experience, compared to that of 17-year-olds. We conjectured that it may be the case that, in children, the experience of training on any sequence of finger opposition movements affects the performance of the individual elements of the sequence rather than the syntactic rule which has been implicated in the learning of the task by adults [1]. There is additional evidence indicating differences in the consolidation of procedural knowledge before and after puberty [27]. Our current results show that it is unlikely that before adolescence children continue to represent the trained movement sequence as individual movements rather than as a specific ordered movement set. A single session of training on the order of 200 iterations of the movement sequence, sufficed to generate (by 48 hours post-training) a sequence-specific but effector independent representation of the set of trained movements in all age groups tested. There were no general improvements of the individual, component movements that comprised the sequences in the younger age groups; rather, processes resulting in the “chunking” and co-articulation of individual movement elements into specific movement sequences are likely to subserve movement sequence learning in children as in adults [2]–[5], [28].
